# Economic Recession and Emergence of an HIV-1 Outbreak among Drug Injectors in Athens Metropolitan Area: A Longitudinal Study

**DOI:** 10.1371/journal.pone.0078941

**Published:** 2013-11-12

**Authors:** Dimitrios Paraskevis, Georgios Nikolopoulos, Anastasios Fotiou, Chrissa Tsiara, Dimitra Paraskeva, Vana Sypsa, Marios Lazanas, Panagiotis Gargalianos, Mina Psichogiou, Athanasios Skoutelis, Lucas Wiessing, Samuel R. Friedman, Don C. d. e. s. Jarlais, Manina Terzidou, Jenny Kremastinou, Meni Malliori, Angelos Hatzakis

**Affiliations:** 1 National Retrovirus Reference Center, Department of Hygiene, Epidemiology and Medical Statistics, Medical School, University of Athens, Athens, Greece; 2 Hellenic Center for Disease Control and Prevention, Athens, Greece; 3 Greek REITOX Focal Point of the EMCDDA at the University Mental Health Research Institute (UMHRI), Athens, Greece; 4 3^rd^ Department of Internal Medicine, Hellenic Red Cross Hospital, Athens, Greece; 5 1^st^ Department of Internal Medicine, Athens General Hospital “G. Gennimatas”, Athens, Greece; 6 1^st^ Department of Propaedeutic Medicine, Athens University Medical School, Laiko Hospital, Athens, Greece; 7 5^th^ Department of Medicine and Infectious Diseases, “Evangelismos” General Hospital, Athens, Greece; 8 European Monitoring Centre for Drugs and Drug Addiction (EMCDDA), Lisbon, Portugal; 9 National Development and Research Institutes (NDRI), New York, New York, United States of America; 10 Beth Israel Medical Centre, New York, New York, United States of America; 11 Organisation Against Drugs (OKANA), Athens, Greece; Chinese Academy of Sciences, Wuhan Institute of Virology, China

## Abstract

**Background:**

During 2011, a dramatic increase (1600%) of reported HIV-1 infections among injecting drug users (IDUs) was noted in Athens, Greece. We herein assess the potential causal pathways associated with this outbreak.

**Methods:**

Our study employed high resolution HIV-1 phylogenetic and phylogeographic analyses. We examined also longitudinal data of ecological variables such as the annual growth of gross domestic product (GDP) of Greece in association with HIV-1 and HCV sentinel prevalence in IDUs, unemployment and homelessness rates and HIV transmission networks in Athens IDUs before and during economic recession (2008–2012).

**Results:**

IDU isolates sampled in 2011 and 2012 suggested transmission networks in 94.6% and 92.7% of the cases in striking contrast with the sporadic networking (5%) during 1998–2009. The geographic origin of most HIV-1 isolates was consistent with the recently documented migratory waves in Greece. The decline in GDP was inversely correlated with annual prevalence rates of HIV and HCV and with unemployment and homelessness rates in IDUs (all p<0.001). The slope of anti-HCV prevalence in the sentinel populations of IDUs and in “new” drug injectors was found 120 and 1.9-fold (p = 0.007, p = 0.08 respectively) higher in 2008–2012 (economic recession) compared with 2002–2006. The median (25th, 75th) size of transmission networks were 34 (12, 58) and 2 (2, 2) (p = 0.057) in 2008–2012 and 1998–2007, respectively. The coverage of harm reduction services was low throughout the study period.

**Conclusions:**

Scaling-up harm reduction services and addressing social and structural factors related to the current economic crisis should be urgently considered in environments where HIV-1 outbreaks may occur.

## Introduction

Human immunodeficiency virus (HIV) infection remains a significant public health problem globally with 34 million HIV estimated to be infected people worldwide (http://www.unaids.org). Injecting drug users (IDUs) or people who inject drugs (PWID) represent up to 10% of the HIV infected population worldwide, but this proportion rises to 30% outside Africa [1]. In the European Union, IDUs account for a relatively small proportion (4% in 2010) of the annual number of newly diagnosed reported HIV cases with the majority of countries reporting less than 1 per 100 000 population among IDUs [2]. Until the beginning of 2011 the HIV-1 epidemic in Greece was concentrated in men who have sex with men (MSM) with only a few sporadic cases among IDUs [3]. During the first months of 2011 an unprecedented upward shift in the number of newly diagnosed IDUs was observed [4], [5] in an environment where prevention and harm-reduction services such as opioid substitution treatment (OST) and needle syringes programs (NSP) were available albeit with rather than in low coverage [6]. Prior to the outbreak, the HIV-1 epidemic in Greece was highly divergent in terms of molecular epidemiological patterns [7], [8].

Before 2011, drug injectors in Greece were already marginalized facing discrimination and quite frequently oppression by law enforcement services. They were engaged in risky behaviors for acquiring or transmitting viral pathogens even though they were infected only with hepatitis C virus (HCV) [3]. The perilous practices were occurring in an environment of limited harm reduction measures as mentioned above [6]. The HIV surveillance system was based on the mandatory reporting of HIV cases and on calculating prevalence estimates among IDUs approaching treatment facilities. Substantial changes in patterns of testing and reporting have not been observed while ushering in the period of HIV explosion. However, the salient feature of the HIV outbreak among IDUs in Greece is that it emerged in the context of an enormous economic downturn that has resulted in massive impoverishment, striking unemployment rates, extensive homelessness in the marginalized communities and negative health consequences for the entire population [9], [10], [11].

Given the rapid and extensive HIV spread among IDUs in Greece, it was imperative to study as thoroughly as possible the characteristics and the potential causes of the outbreak. We studied longitudinally the patterns of HIV-1 spread in IDUs in order to assess the existence of local transmission networks (phylogenetic clusters), their temporal distribution, the origin of circulating HIV-1 sequences (phylogeography) and to identify potential founders or index cases. We ecologically correlated longitudinal sentinel virological data and HIV-1 molecular characteristics of IDUs with temporal trends of the annual growth of gross domestic product (GDP) in view of the current economic recession. Our analysis aimed to identify potential causal pathways to better tailor future preventive actions.

## Methods

### Ethics Statement

The demographic and virologic parameters, the dates of diagnosis and the nationality of cases were recorded in an electronic anonymised database provided by the Hellenic Center for Disease Control and Prevention (HCDCP) after anonymous matching that has been approved by the Hellenic Data Protection Authority. Specifically the matching of data was performed using birth dates, initials and gender. For the purpose of the current study, no informed consent was needed.

### Study Population

Surveillance data were retrieved from the HIV registry at the HCDCP. To identify the origin and patterns of spread for HIV-1 strains among IDUs, phylogenetic analyses were performed on HIV-1 sequences from all IDUs collected from 1998 to 2012 (n = 282). More specifically 76 specimens were collected in 1998–2009, 12 specimens were collected in 2010, 112 samples were collected in 2011 and 82 samples between January-May 2012. The 112 samples collected in 2011 represent 43% (112/260) of the reported HIV positive IDU cases in that year, but the percentage ranged from 11.8% in 2006 to 100% in 2004 (**Table S2 in File S1**). Molecular HIV-1 data of IDUs were nested and compared with sequence data from the Hellenic HIV-1 Sequence Database (HHSD) (**Methods in File S1**). All but 34 IDUs were targeted for sequencing for the purpose of drug resistance testing. Additionally at the early stage of the outbreak in 2011, samples from 34 IDUs were randomly collected after their diagnosis in collaboration with the Hellenic Center of Diseases Control and Prevention. Although the possibility of selection bias cannot be excluded, IDUs analyzed in this study were representative for gender and ethnicity during 2000–2010, 2011 and 1/1-31/5/2012 as compared with the total number of IDUs reported in the corresponding time periods. The only difference was for last period where IDUs with Greek origin were overrepresented in our sample (82.9% in our study versus 76.3% for the total reported IDUs).

Additional anonymous sentinel virological data such as prevalence of anti-HIV-1, prevalence of antibodies to hepatitis C virus (anti-HCV) and information on socioeconomic indicators such as unemployment rate and homelessness rate of IDUs accessing treatment were collected from the Greek REITOX Focal Point of the EMCDDA in addition to data on the coverage of harm reduction services such as OST and NSP. Trends in HCV prevalence in IDUs, and especially prevalence in newly initiated (“new”) IDUs (injecting less than 2 years), were analyzed as an incidence proxy for HCV, which may reflect changes in injecting risk behaviors [6], [12], [13]. HIV-1 sequences from IDUs before 2011 have been previously published [7]. Sequences from recently reported HIV-1 cases have been submitted to the sequence database (GenBank accession numbers: KF444838–KF444893).

### Hellenic HIV-1 Sequence Database (HHSD)

The HHSD is comprised by sequences [protease (PR) and partial reverse transcriptase (RT)] from 2,327 anonymised HIV-1 infected individuals across Greece as described previously [8].

### Phylogenetic and Phylodynamic Analyses

Phylogenetic trees were inferred by maximum likelihood method. Only grouped sequences from IDUs sampled from Greece that received Shimodaira-Hasegawa (SH) values higher than >0.95 were considered as IDU phylogenetic clusters (local transmission networks). Phylodynamic analyses were performed using Bayesian method. Molecular clock analyses revealed the time to most recent common ancestor (tMRCA). The tMRCA of a phylogenetic cluster is the time since the founding of the cluster or the time since the first transmission that led to the formation of the observed clustered infections. It also provides the upper bound for the time of secondary infections within the members of the cluster.

### Economic Data

Information on the annual growth rate of GDP in Greece was obtained from the website of the European Statistical Agency (Eurostat; http://epp.eurostat.ec.europa.eu/portal/page/portal/eurostat/home/). GDP is a measure of economic activity, defined as the value of all goods and services produced minus the value of any goods or services used in the creation process. For measuring the growth rate of GDP in terms of volumes, the GDP at current prices are valued in the prices of the previous year and the calculated volume changes are imposed on the level of a reference year. The computation of the annual growth rate of GDP allows comparisons of the dynamics of economic development both over time and between economies of different sizes. For this analysis the GDP of Greece was considered as fairly representative of Athens GDP.

Economic recession was defined as “a significant decline in economic activity spread across the economy, lasting more than a few months, normally visible in real GDP, real income, unemployment, industrial production and wholesale retail sales. A recession begins after the economy reaches a peak of activity and ends as the economy reaches its trough” [14]. Economic recession for this study was defined by the period 2008–2012 although 2012 was not the end of the current crisis.

### Statistical Analysis

The potential relationship between the reporting rates of newly diagnosed HIV-1 infection in IDUs and the annual growth rate of GDP was examined applying negative binomial regression models with robust variance estimates. Simple linear regression models with robust variance estimates were used to assess the association of the prevalence estimates of HIV-1 (logged), hepatitis C virus infection (antibodies) and the unemployment and homelessness (including those who temporarily lack stable accommodation) rates in IDUs in Athens (capital city of Greece) with the growth rate of GDP. Using weighted least squares regression (weighted by the number of IDUs tested per year), we estimated the slopes and their 95% confidence intervals (CI) of the change of the annual prevalence rates of HCV in all IDUs and in new drug injectors separately for the time periods 2002–2006 and 2008–2012. These slopes represent the change in HCV prevalence per 100 IDUs per year. Associations between categorical variables were assessed using the chi-squared test. All statistical analyses were conducted in STATA (v.12.0).

### Role of Funding Source

No funding organization had any role in the writing of the report or the decision to submit for publication. The corresponding author had full access to all data in the study and final responsibility for the decision to submit for publication.

## Results

### HIV-1 Surveillance in Greece

**Figure 1:**
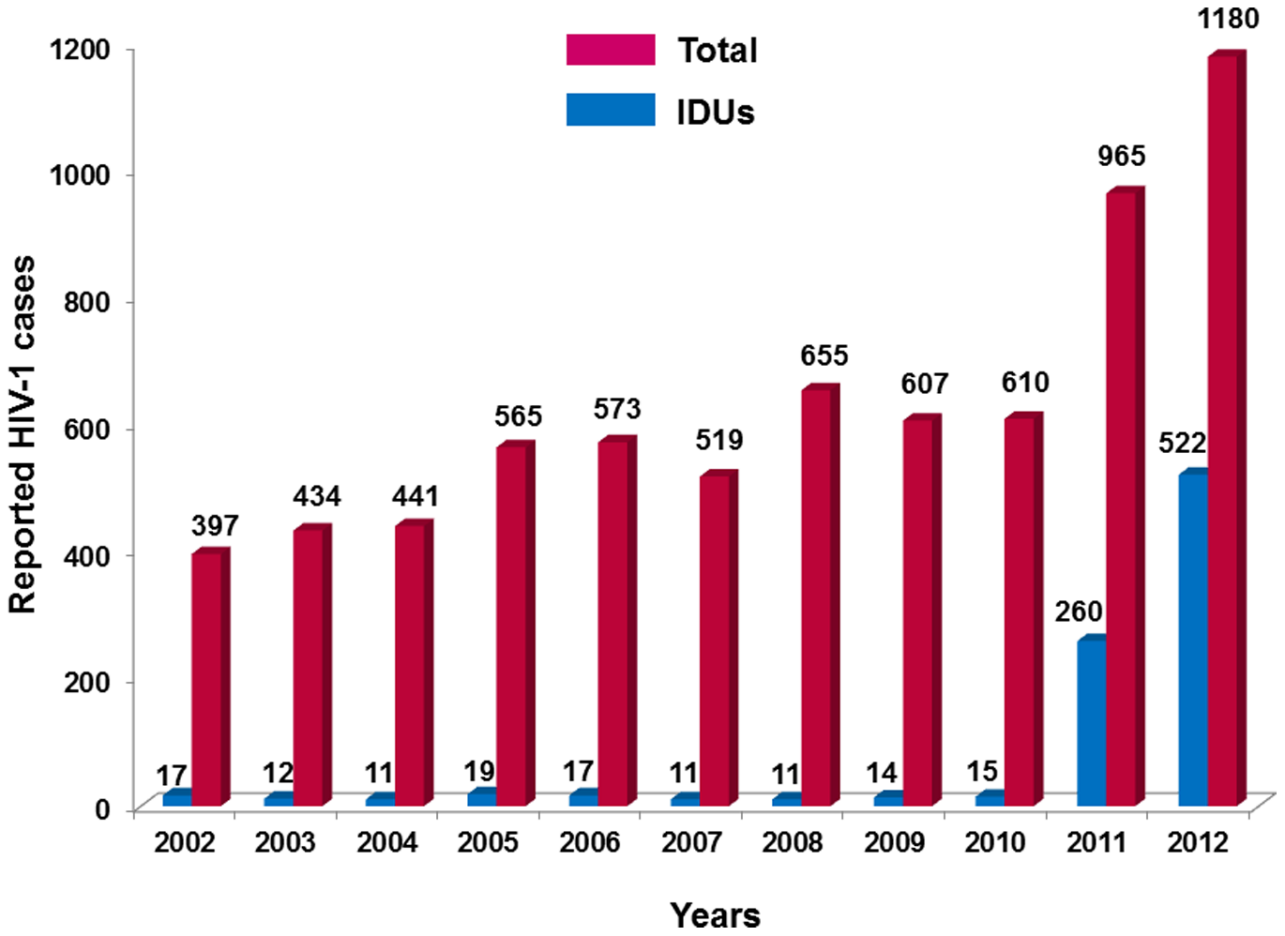
Number of reported HIV-1 cases among injecting drug users (IDUs) (blue) and the total population (red) over different years.

**Figure 2:**
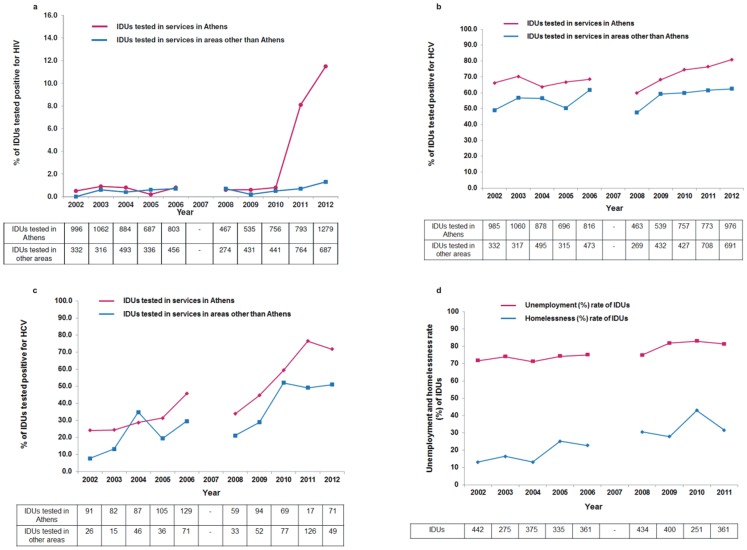
a Sentinel anti-HIV prevalence in IDUs entering drug treatment or accessing low-threshold services during the years 2002–2012 in Athens and areas other than Athens. b Sentinel anti-HCV prevalence in injecting drug users (IDUs) entering drug-related treatment or accessing low-threshold services during 2002–2012 in Athens and areas other than Athens. c Sentinel anti-HCV prevalence in IDUs with injecting history of less than 2 years (“new” IDUs) entering drug-related treatment or accessing low-threshold services during 2002–2012 in Athens and areas other than Athens. d Unemployment and homelessness rate drug injectors during 2002–2011 in Athens. Data were not collected in 2007.

During 2002–2010, between 11 and 19 IDU cases were reported annually among new HIV-1 reports representing 2–4% of the total HIV-1 reports per year. During 2011, the number of HIV-1 positive IDUs increased sharply to 260 (2.41 cases per 100,000 population), accounting approximately for 27% of all recorded HIV-1 infections that year (Figure 1) and representing a 16-fold increase compared with 2010. Most of HIV-1 positive IDUs reported in 2011 were males (n = 211, 81.2%) and of Greek nationality (n = 211, 81.2%) (Table S1 **in File S1**). Longitudinal sentinel prevalence data of anti-HIV-1 demonstrate that HIV-1 transmissions were limited to Athens Metropolitan area (Figure 2a).

### The Molecular Epidemiology of the IDU Epidemic in Greece

The prevalence of HIV-1 subtypes and recombinant forms in IDUs and in the total population of HIV-1 infected individuals sampled from 1998 to 2009 were similar (p = 0.12) (Table 1) [8]. However, the prevalence of HIV-1 clades differed significantly between the populations of IDUs sampled during 1998–2009 and 2010–2012 (p<0.001). Notably, more than half (64.6%) of the IDUs in the years 2010–2012 were classified as CRF35_AD and CRF14_BG, and these clades were not detected in the population previously (1998–2009) (Table 1).

**Table 1:** Prevalence of HIV-1 subtypes and recombinant forms.

Subtypes (n, %)Populations	A	CRF35_AD	B	CRF14_BG	CRF02_AG	Others	Total	
Total sampled HIV-1 infected population (1998–2009)	572 (24.6%)	0	1,396 (60.0%)	0	44 (1.9%)	315 (13.5%)	2,327	
IDUs (1998–2009)	22 (28.9%)	0	43 (56.6%)	0	4 (5.3%)	7 (9.2%)	76	*p* 1 = 0.12
IDUs (2010–2012)	23 (11.2%)	58 (28.2%)	41 (19.9%)	75 (36.4%)	1 (0.5%)	8 (3.9%)	206	*p* 2<0.001

IDUs: Injecting Drug Users; CRF: Circulating Recombinant Forms (CRFs).

1 Comparison of the prevalence of subtypes and CRFs between the total sampled HIV-1 infected population (1998–2009) and the IDUs (1998–2009).

2 Comparison of the prevalence of subtypes and CRFs between the IDUs sampled during 1998–2009 and 2010–2012.

### Patterns of HIV-1 Spread in IDUs

To describe the patterns of HIV-1 spread in IDUs in different time periods we performed separate phylogenetic analyses for each of the IDU clades (subtype A and CRF35_AD, B, G and CRF14_BG, CRF04_cpx and unclassified) sampled from 1998 to 2012 using sequences from the HHSD.

The profile of the epidemic spread in IDUs exhibited significant time trends since almost all infections (93.8%) in IDUs from 2011–2012 were clustered compared to 41.7% in 2010 and only 5.3% before 2010 (Table 2, Table S2 in File S1). We identified 8 IDU specific clusters or local transmission networks during 1998–2012 consisting of 75 (CRF14_BG), 58 (CRF35_AD), 34 (subtype B), 12 (subtype A), 2 (A_FSU_), 2 (CRF02_AG), 2 (subtype B) and 2 (subtype B) IDUs (Table 3). All IDU samples reported during 2011–2012 were grouped in the four larger phylogenetic clusters and 39% of them were classified as CRF14_BG (Table 3).

**Table 2:** Patterns of HIV-1 spread in IDUs.

HIV spread (n,%) Populations	Clustered HIV-1 infections	Non-clustered HIV-1 infections	Total	
**IDUs (1998–2009)**	4 (5.3%)	72 (94.7%)	76	
**IDUs (2010)**	5 (41.3%)	7 (58.3%)	12	*p* * <0.001
**IDUs (2011)**	106 (94.6%)	6 (5.4%)	112	*p* * <0.001
**IDUs (2012)**	76 (92.7%)	6 (7.3%)	82	*p* * <0.001

IDUs: Injecting Drug Users.

* Comparisons of each group with the IDUs samples during 1998–2009.

**Table 3:** Characteristics of phylogenetic clusters among injecting drug users (IDUs).

IDU phylogeneticcluster		IDUs percluster (N_0_)	Samplingperiod	Geographicalorigin	Nationality ofpotential founder	tMRCA(95% HPD)
ID	Clade					
1	CRF14_BG	75	2010–2012	W.Europe	Bulgaria	2009 (2008–2010)
2	CRF35_AD	58	2011–2012	Afghanistan-Iran	Iran	2009 (2007–2011)
3	B	34	2011–2012	Greece	Greece	2008 (2003–2010)
4	A	12	2011–2012	Greece	Greece	2010 (2009–2011)
5	A_FSU_	2	2010	FSU	NA	2008 (2005–2010)
6	CRF02_AG	2	2005 and 2010	NA	NA	2005 (2003–2005)
7	B	2	2002 and 2010	NA	NA	1996 (1986–2001)
8	B	2	2002 and 2003	NA	NA	1985 (1972–1995)

**FSU:** Former Soviet Union.

**tMRCA:** Time to most recent common ancestor.

**HPD:** Higher Posterior Density.

**NA:** Not Available.

### Phylogeography of HIV-1 Sequences in IDUs

The most similar sequences for subtype A were from Iran and Afghanistan previously assigned as CRF35_AD [15], while for subtype G were from Portugal and Spain belonging to CRF14_BG. The outbreak-specific subtype B cluster (N = 34) was part of the larger subtype B patient groups from Greece (data not shown). Similar results were found for the clade of subtype A (12 sequences) (Figure S1), while the other one (2 sequences) fell within the branch of strains (A_FSU_) causing the large IDUs epidemic across former Soviet Union (FSU) countries (Figure S1). Smaller clusters of subtype B and CRF02_AG were comprised of isolates from diverse geographic areas, therefore their origin was not identified (Table 3).

### Phylodynamic Analyses

**Figure 3:**
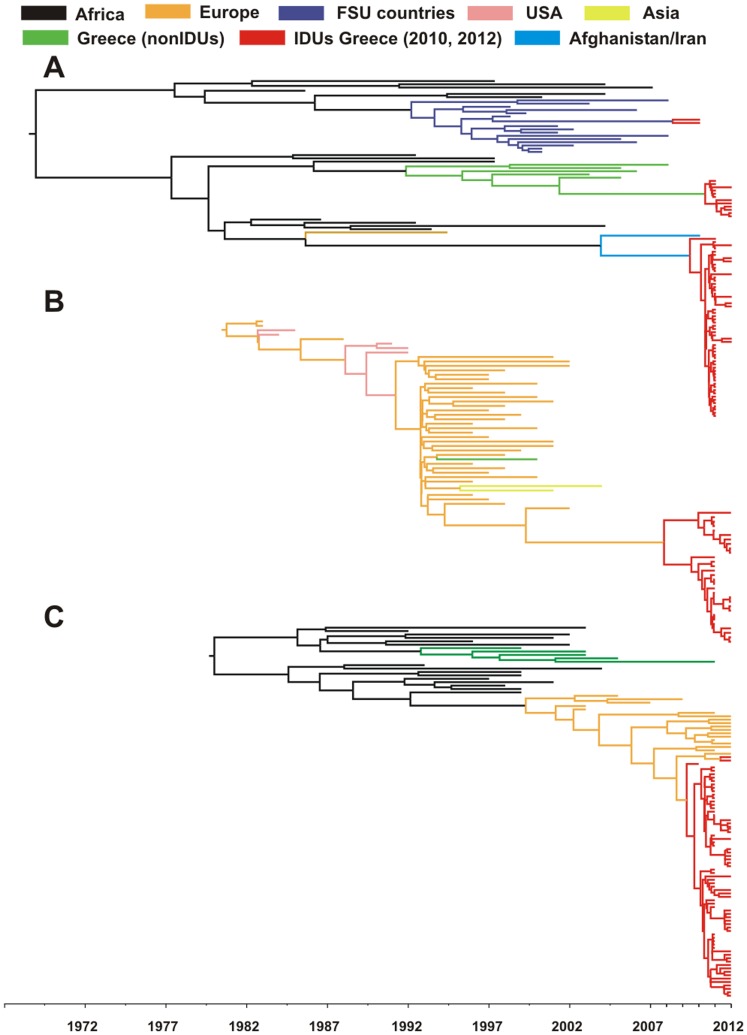
Molecular clock analyses of HIV-1 sequences from: A. subtype A and CRF35_AD B. subtype B and C. subtype G and CRF14_BG sampled from different geographic areas and Greece.

The tMRCA of IDU groups was dated in 2009 for the CRF14_BG and CRF35_AD (clusters 1 and 2) [95% Higher Posterior Densities (HPD): 2008–2010 and 2007–2011, respectively] (Figure 3A and C), in 2008 for cluster 3 [95% HPD: 2003–2010] (Figure 3B), in 2010 and 2008 for clusters 4 and 5 [95% HPD: 2009–2011 and 2005–2010, respectively], in 2005 for cluster 6 [95% HPD: 2003–2005], in 1996 for cluster 7 and in 1985 [95%HPD: 1986–2001] for cluster 8 [95% HPD: 1972–1995] (Table 3). Most of the IDUs specific clades coalesce at the very recent time period of approximately of 1–2 years, which is compatible with the epidemiological surveillance findings. Potential founders of the major sub-outbreaks have been identified (Figure S2).

### Ecologic Associations with GDP and Economic Recession

**Figure 4:**
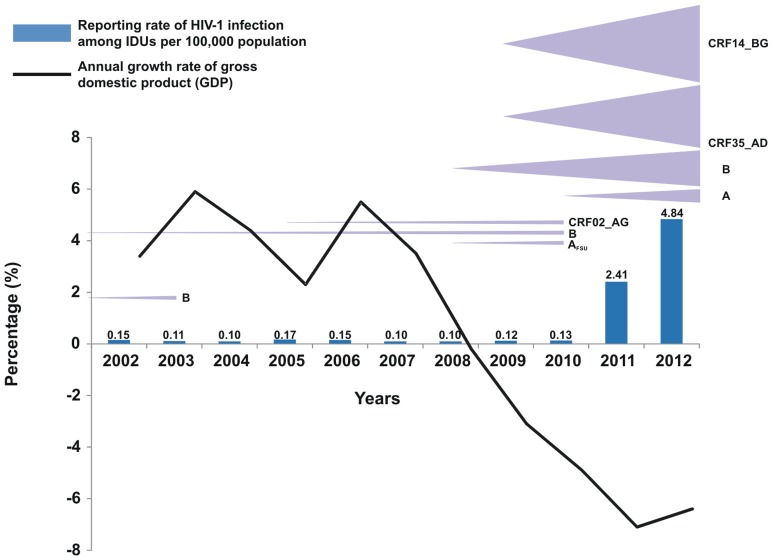
Plot of the reporting rate of HIV-1 infection among injecting drug users (IDUs) per 100,000 of population in relation with the annual growth rate of gross domestic product (GDP). IDU clusters are shown by triangles proportional to the number of sequences. The time to most recent common ancestor (tMRCA) for the different outbreaks is denoted at the top of each triangle. The tMRCA for both subtype B clusters 1 and 2 was estimated before 2002 not shown in figure.

As shown in Figure 4, the Greek economy entered a phase of continuous recession in 2008 and the GDP has contracted considerably during the years 2008–2012. The number of HIV-1 infections reported in Greece the last decade seems to be inversely correlated with the annual growth rate of GDP. Negative binomial regression models with robust standard errors revealed a statistically significant negative association between the annual change of GDP in Greece and the reporting rate of HIV-1 infection [Reporting rate ratio (RRR) for one unit increase in the annual growth rate of GDP: 0.95, 95% CI: 0.92–0.97; p<0.001] or with the reporting rate of IDUs in HIV-1 cases in (RRR in IDUs for one unit increase in the annual growth rate of GDP: 0.78, 95% CI: 0.71–0.85, p<0.001). Using linear regression analyses, we also found that the yearly change rate of GDP was strongly and negatively associated with the homelessness rate (b = −1.73, r = −0.82, p = 0.01) and, to a lesser degree, with the unemployment rate in Athens’ drug injectors (b = −0.97, r = −0.79, p = 0.01) (Table 4) (Figure 2d). In addition, the change in GDP growth per year was significantly and negatively associated with the HIV-1 prevalence (logged values) among drug injectors who approached treatment facilities in Athens (b = −0.16, r = −0.65, p = 0.05), with the prevalence of HCV antibodies in the same population (b = −0.82, r = −0.66, p = 0.02), and with a proxy indicator of HCV incidence in IDUs i.e. with the prevalence of HCV antibodies among “new” drug injectors in the Greek capital city (b = −3.33, r = −0.87, p = 0.001) (Table 4).

**Table 4:** Univariable ecological associations between the annual growth rate of Gross Domestic Product (GDP) in Greece and various dependent variables for the period 2001–2012.

Dependent variable	Model^1^	Coefficient^2^	95% CI	P-value
Reporting rate of HIV-1 infection in drug injectors	Negative binomialregression	0.78	(0.71, 0.85)	<0.001
Prevalence of HIV-1 infection^3^ among drug injectors in Athens^4^	Linear regression	−0.16	(−0.32, 0.00)	0.05
Prevalence of HCV infection (antibodies) among drug injectors in Athens^4^	Linear regression	−0.82	(−1.47, −0.17)	0.02
Prevalence of HCV infection (antibodies) among new drug injectors in Athens^4, 5^	Linear regression	−3.33	(−4.93, −1.73)	<0.001
Homelessness rate among drug injectors in Athens^6^	Linear regression	−1.73	(−2.87, −0.59)	0.01
Unemployment rate among drug injectors in Athens^6^	Linear regression	−0.97	(−1.53, −0.41)	0.01

1 CI: Confidence Interval, HIV: Human Immunodeficiency Virus, HCV: Hepatitis C VirusIn all models, robust estimates of variance have been used.

2 For the negative binomial regression model, the coefficient has been exponentiated corresponding to the reporting rate ratio of HIV-1 infection in drug injectors for one unit increase in the annual growth rate of GDP [i.e. for 1% increase of GDP each year, the reporting rate of HIV-1 infection in drug injectors reduces by 1–0.78 = 0.22 or 22%).

3 The prevalence of HIV-1 infection has been logged (natural logarithm).

4 Estimated among drug injectors who accessed drug treatment -or low-threshold services in Athens. Data were not available for 2001 and 2007.

5 “New” drug injectors: IDUs with injecting history of less than 2 years.

6 Estimated among drug injectors who contacted treatment services or low-threshold facilities in Athens. Data for 2007 and 2012 were not available.

Around the time of economic recession (2008–2012), 86.6% of the infections in IDUs were clustered compared to 6.1% during the years 1998–2007 (p<0.001) (Table S2 **in File S1**). The median (25th, 75th) size of transmission networks were 34 (12, 58) and 2 (2, 2) (p = 0.057) in 2008–2012 and 1985–2007, respectively.

The temporal trends of sentinel anti-HCV prevalence in Athens Metropolitan Area suggested a relatively stable prevalence in the population of IDUs during 2002–2006 and an increase during 2008–2012 (data for 2007 not available) (Figure 2b). According to the slopes estimated in the two time periods, the prevalence increased by 4.8% per year in 2008–2012 (p = 0.006) as compared to 0.04% per year in 2002–2006 (p = 0.97) (difference in slopes (95% CI): 4.7 (1.9, 7.6), p = 0.007). Among “new” IDUs, increasing HCV prevalence was observed throughout the years 2002–2012, although the slope of anti-HCV prevalence became steeper during the years 2008–2012 (Figure 2c). The prevalence increased by 9.9% per year in 2008–2012 (p = 0.007) as compared to 5.2% per year in 2002–2006 (p = 0.04) (difference in slopes (95% CI): 4.6 (−0.6, 9.9), p = 0.08). Thus, the slopes representing the change in anti-HCV prevalence per year, as proxy of injecting risks, were found to be 120-fold and 1.9-fold higher in 2008–2012, as compared to 2002–2006, in all IDUs and in “new” IDUs, respectively.

### Coverage of Prevention Services

Despite significant increases in 2011 and 2012 (Figure S3a, 3b), the coverage of OST and NSP had been low until 2010. In particular, in the period 2002–2010, the estimated number of distributed/exchanged needles/syringes per IDU per year in Athens Metropolitan Area was below 20 (note that an average heroin injector may inject some 1000 times or more per year and this number may significantly increase when stimulants are also injected) [16], while the proportion of problem opioid users in OST remained below 30%^6^ and waiting time for OST entry extended to over five to eight years.

## Discussion

Over the last 20 years, Greece has experienced a massive influx of economic and other migrants from countries in Southeastern/Central Europe, the Middle East, Asia and Africa, with the majority of them being concentrated in Athens Metropolitan Area [17]. These migratory waves are likely to influence the epidemiological patterns of many infectious diseases, including HIV-1 [18]. For reasons that also bear on economic crisis, the number of registered foreigners has dropped in the period 2009–2012 from more than 600,000 in 2009 to almost 450,000 in 2012 (i.e. 28% decrease). The number of residence permits has also been reduced by more than half during the same period [19]. The latter is thought to be associated with employment crisis among immigrant populations–unemployment in this population increased from 7% in 2008–9 to 28% in 2011. In fact the origin of the second largest cluster (CRF35_AD) was initially reported as spreading among IDUs in Afghanistan and Iran [15], and the origin of the largest cluster has been attributed to strains originated in Southwestern Europe. The two other clusters originated from Greece. To our knowledge this is the first introduction of HIV-1 specific strains circulating in the major heroin producing area of Afghanistan into Europe. In the two major sub-outbreaks, the potential founders were identified as connected with the migrant IDU population as part of the greater IDU transmission network in Athens. Phylogenetic analysis suggested also that other migrant IDUs have been infected after their arrival in Athens.

The reporting rates HIV-1 infection in IDUs, the prevalence of HIV-1 in IDUs accessing treatment services in Athens and the annual prevalence estimates of anti-HCV antibodies in this population (both for all drug injectors tested and for those who had started injection the last 2 years) were inversely correlated with the annual change of GDP in Greece. Although no changes in testing policy have been noted, the number of HIV tests conducted has been nonetheless increased in the period following the detection of HIV outbreak in 2011 [20]. Increases in testing were due to increases in public awareness but the scaling-up of OST (in late 2011 and throughout 2012) and the implementation of ARISTOTLES a STTR strategy program implemented in Athens in the later part of 2012 have played a critical role to the observed increases. Moreover phylogenetic analyses revealed that sequences from IDUs during the outbreak were genetically similar suggesting the recent nature of the HIV-1 epidemic among them.

In addition, the present study showed that both, homelessness and unemployment rates among IDUs in Athens were inversely related to the GDP growth rate suggesting that the decline in GDP has most likely affected the social-economic conditions of IDUs living in the capital city.

Doubtless, these are ecological associations and biases cannot be excluded although ecological studies may provide information that individual level studies cannot [21]. Previous research has shown, however, that economic and sociopolitical transitions have helped unleash HIV epidemics in some countries [9], [22], [23]. It should be noted that the outbreak cannot be attributed to a single random HIV-1 founder event since 5 “successful” independent HIV-1 IDU clusters during the period 2008–2012, after the initiation of the economic downturn, were observed. The HIV-1 infections within 5 transmission networks during 2008–2012 resulted in 86.6% of clustered HIV-1 transmissions, while the other 3 transmission networks during 1985–2007 had resulted in only 6.1% of clustered HIV-1 transmissions.

The yearly increase of sentinel anti-HCV prevalence during 2008–2012, in comparison with 2002–2006, in all and “new” IDUs, suggests acceleration of increasing risk behaviors after the initiation of economic recession. Low-threshold data in Athens show no changes in current sharing of injecting equipment between 2008 and 2011, but show increases in the abuse of cocaine, and other stimulants, and increases in current injecting. Increases in the abuse of cocaine among all, immigrants, unemployed and homeless IDUs in Athens have been observed also in treatment demand data [20]. No data are available regarding trends in the exposure to commercial sex-work among IDUs.

The provision of HIV-related prevention or harm-reduction services to IDUs in Greece has remained, with some fluctuations, at low to very low levels during the last decade [24]. It is thus likely that some socially marginalized groups, especially the undocumented immigrant IDUs had no access to OST. Indeed in an ongoing study of 1,404 IDUs in Athens, 33.6% of the participants reported having been homeless within the past year and 15.1% were migrants [25]. The proportion of migrants who have ever entered OST was 8.5% as compared to 26.4% of Greek participants suggesting – if not an inequitable distribution of OST services– structural barriers in accessing harm reduction services by vulnerable populations in Athens.

**Figure 5:**
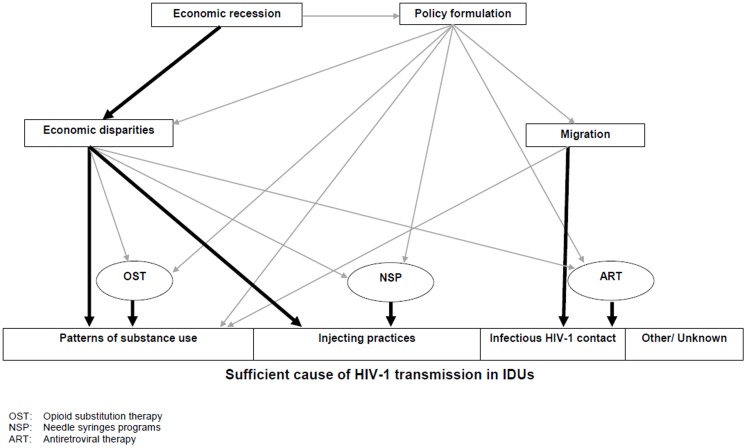
Working hypothesis for the HIV-1 outbreak in Athens Metropolitan Area. Bold arrows indicate causal pathways suggested in the current study. Thin arrows indicate other possible causal pathways.

Figure 5 depicts a working hypothesis on the causal pathways of the current outbreak based on causal diagrams and the sufficient component cause model [26], [27]. The potential pathways of an HIV-1 outbreak are complex^15^ but this study suggests a simpler hypothetical set of causal pathways for the Greek outbreak: First, economic recession led to increased socioeconomic disparities and difficulties among injectors in Athens leaving a significantly higher proportion of them jobless and another significant proportion without accommodation. Second, this then facilitated the increase development of large transmission networks of injectors. Third, because injecting equipment was scarce, IDUs shared paraphernalia with large numbers of other injectors within short time periods (rapid partner change and high concurrency), as suggested by increased HCV transmission among “new” IDUs. Fourth, migrant IDUs, being also themselves victims of the economic and/or social instability in their country that forced them to move, introduced new HIV-1 strains into these large sharing networks of susceptible drug injectors. Fifth, by implication, an outbreak with very rapid transmission occurred. It seems that macro-level changes such as the recent economic crisis - directly or indirectly - through the increasing socioeconomic disparities and difficulties such as unemployment, extreme poverty, homelessness, stigma, discrimination and social isolation of IDUs and through the budgetary constraints and poor policies for financing prevention and treatment can be translated to heightened risk behaviors on the individual level and impaired public health response on the population level, which can then jointly facilitate HIV-1 transmissions [28] in an environment with already low coverage of prevention services [6]. Even in the absence of economic and social turbulence, HIV-1 disproportionately affects key populations such as IDUs, sex workers, MSM, homeless people and migrants from countries with generalized HIV epidemics. In particular, migrant IDUs represent a highly vulnerable group with many barriers to access prevention, care and treatment services [29].

The Athens HIV-1 outbreak in IDUs can be regarded as a natural experiment providing insights on the complex cascade of events by which distal causes such as economic recession and migratory waves challenge public health action targeted at HIV prevention [30]. These factors may affect proximal causes such as risk behaviors resulting in HIV-1 transmissions and full- blown outbreaks. These findings indicate that addressing social and structural factors may play a significant role in the control of HIV-1 in IDUs and other vulnerable populations [31], [32] We have also shown that timely conducted high resolution molecular typing during outbreaks in connection with epidemiological and behavioral surveillance data analyses may contribute to identify transmission networks and populations at risk [33], [34] and, as such, will contribute to focus future preventive services. The population at risk is reported in Athens Metropolitan Area. The involvement of migrant IDUs from Afghanistan was a post-hoc finding not included in our hypotheses. However one major limitation of our study is its ecological nature. The conclusions of the present study are tested in a large prospective individually based study (ARISTOTLE program) that is currently in progress.

## Supporting Information

Figure S1 Part of phylogenetic tree for subtype A sequences sampled from different areas [Africa, Albania, other European countries, former Soviet Union (FSU) areas, Afghanistan/Iran and Greece]. Different colors were used for the non-injecting drug users (IDUs) and IDUs sampled before and after 2010 from Greece.
(TIF)

Figure S2 Partial dated phylogenetic trees showing the ethnic origin of the individuals from whom HIV-1 sequences were sampled. For two sub-outbreaks (CRF35_AD and CRF14_BG) the potential founders were non-Greek nationals, while for subtype A and subtype B (A and C) the potential founders were nationals.
(TIF)

Figure S3 **a** Trends in the estimated number of syringes distributed/exchanged through specialized programmes per estimated injecting drug user (IDU) in the city of Athens (NSP coverage, 2004–2011). **b** Opioid substitution treatment (OST) coverage per estimated problem drug user (addicted to opioids) during the years 2002–2011 [10]. Data were not collected in 2007.
(TIF)

File S1 Supporting files.
(DOC)
