# Supplementary figures and images for: Economic Recession and Emergence of an HIV-1 Outbreak among Drug Injectors in Athens Metropolitan Area: A Longitudinal Study
Source: PLoS One. 2013 Nov 12;8(11):e78941. doi: 10.1371/journal.pone.0078941 (PMC3827120; doi:10.1371/journal.pone.0078941)

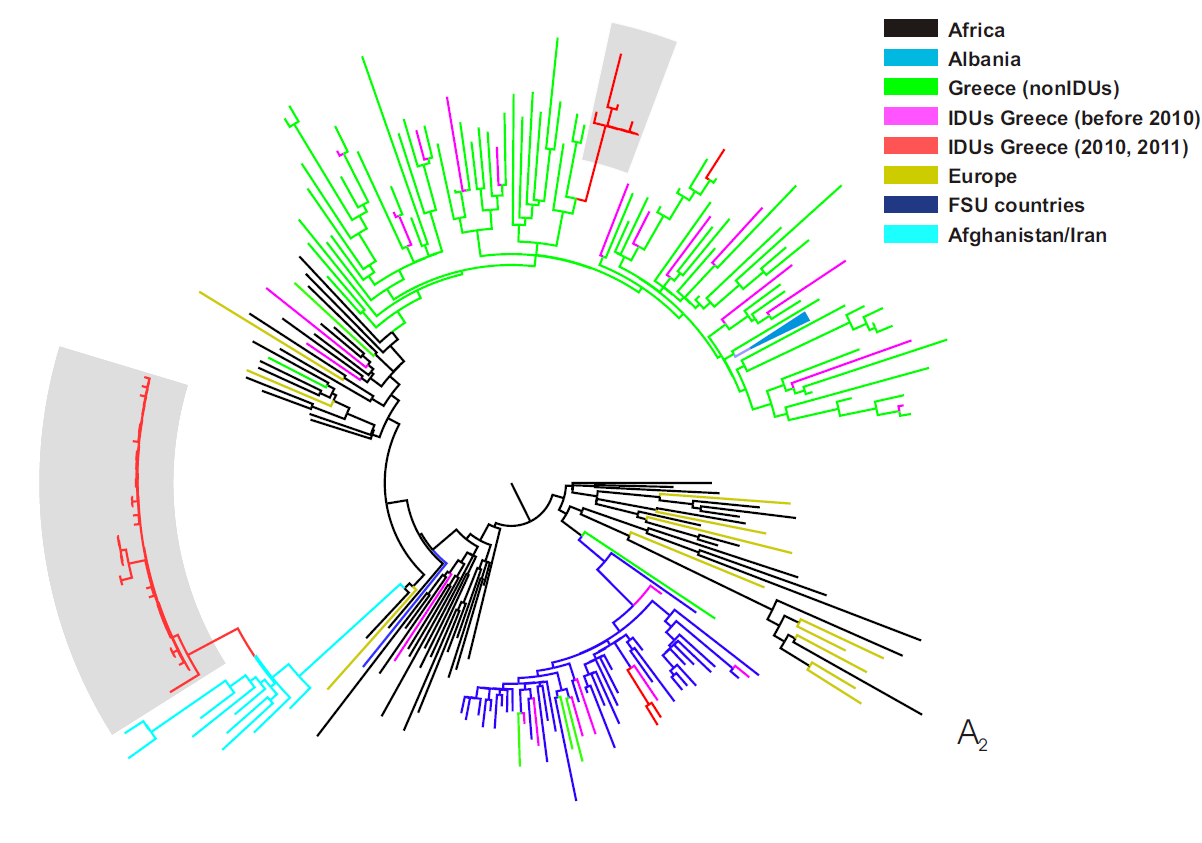

Supplement: Figure S1 — Part of phylogenetic tree for subtype A sequences sampled from different areas [Africa, Albania, other European countries, former Soviet Union (FSU) areas, Afghanistan/Iran and Greece]. Different colors were used for the non-injecting drug users (IDUs) and IDUs sampled before and after 2010 from Greece. (TIF) [file pone.0078941.s001.tif]
