# Supplementary material for: Economic Recession and Emergence of an HIV-1 Outbreak among Drug Injectors in Athens Metropolitan Area: A Longitudinal Study
Source: PLoS One. 2013 Nov 12;8(11):e78941. doi: 10.1371/journal.pone.0078941 (PMC3827120; doi:10.1371/journal.pone.0078941)
Supplement: File S1 — Supporting files. (DOC) [file pone.0078941.s004.doc]

**Supporting Information**

**Methods**

*Hellenic HIV-1 Sequence Database (HHSD)*

HIV-1 sequences were sampled during 1998-2009 from both treated and naïve subjects comprising 20% of the total HIV-1 infected population in Greece since 1984. Demographic and clinical data were available for the majority of patients including the year of HIV diagnosis. The HHSD is representative of the national HIV-1 epidemic according to age, sex and transmission mode.

*HIV-1 subtyping*

HIV-1 PR and partial RT sequences were generated from plasma samples submitted for routine drug resistance testing or from samples collected at the time of HIV-1 diagnosis, using the HIV-1 TRUGENE® Genotyping kit (Bayer, HealthCare) and the ViroSeq™ HIV-1 Genotyping system (Celera Diagnostics, CA, USA). HIV-1 subtypes and recombinant forms were determined by phylogenetic analysis including a set of reference sequences (http://www.HIV.lanl.gov) as described previously and also by means of the COMET HIV-1/2 subtyping tool (v.0.2) (http://comet.retrovirology.lu/).

*Phylogenetic and phylogeographic analyses*

Large phylogenetic trees were estimated with FastTree using the GTR+cat nucleotide substitution model. To avoid bias on the phylogenetic analysis, 37 codons associated with major resistance in PR and RT were stripped from the entire alignment as described previously. Statistical robustness of the clades was assessed using the Shimodaira-Hasegawa (SH) values as implemented in FastTree. Phylogenetic trees were visualized using FigTree version 1.3.1 (http:/tree.bio.ed.ac.uk/software/figtree/). Specifically, phylogenetic trees were generated separately for all IDUs clades [subtypes A, B and G, CRF02_AG and CRF04_cpx]. For 2 IDUs sequences not belonging to any of the previously subtypes or CRFs and showing no evidence of recombination (unclassified), separate phylogenetic analyses was performed in addition with reference sequences from all different subtypes and the most common CRFs (http://www.hiv.lanl.gov).

Similarity searches were performed using the nucleotide-nucleotide Basic Local Alignment Search Tool (BLAST) available from (http://www.ncbi.nlm.nih.gov/BLAST). Final phylogenetic and phylogeographic analyses were performed on the Hellenic HIV Sequence Database and on the 2,715 reference sequences (subtype A and CRF35_AD, n=730; subtype B n=1,542; subtype G and CRF14_BG, n=192; CRF02_AG, n=200; others, n=51) sampled worldwide.

*Phylodynamic analysis*

Phylodynamic analyses were performed using Bayesian method as implemented in BEAST (version 1.5.1). The sequences used for molecular clock analysis were strains from IDUs phylogenetic clusters. Phylodynamic analyses were performed for subtypes A, B and for the recombinant forms CRF35_AD and CRF14_BG. We assembled a data set with IDUs sequences and reference sequences with known sampling dates, available in the Los Alamos database. Phylodynamic analysis was performed using a Bayesian approach as implemented in BEAST (version 1.5.1) using the GTR+G+I nucleotide substitution model. Molecular clock analyses were performed by grouping subtype A and CRF35_AD and, also subtype G and CRF14_BG since the former subtypes are the most closely related groups to these CRFs. We used uncorrelated lognormal relaxed clock model with TipDates and Bayesian skyline plots were used as coalescent tree priors. Two separate Markov chain Monte Carlo (MCMC) runs were made for 10 X 10^6^ generations with a burnin of 10 X 10^5^. MCMC was sampled every 1000 generations. No additional priors were used in the analysis. We assessed convergence and sufficient mixes of the Markov chains (ESS>100) by means of the program Tracer v1.4 (http://tree.bio.ed.ac.uk/software/tracer/). The consensus for each run was inferred by the TreeAnnotator program.

**Results**

*HIV-1 surveillance in Greece*

The total number of HIV-1 infections reported in 2011 was 965, which corresponded to an annual reporting rate of 8∙9 cases per 100,000 population. Compared to 2010, there was a 58% increase. Among reported HIV-1 cases in 2011, the majority were males (n=826, 85.6%) and most of them, in accordance with data from previous years, had been infected through homosexual contact (n=364, 37∙7%). Approximately 18% (n= 178) of the totally reported HIV-1 infections in 2011 were observed in non-Greek nationals. The percentage of non-Greek nationals was slightly lower in IDUs (n=43, 16∙5%) compared to HIV-1 positive individuals infected through other routes (n=135, 19∙2%).

In 2012, there was a new increase (22.3%), compared to 2011, with 1180 cases of HIV-1 infection (10.9 cases per 100,000 population). The majority of reported cases were males (n=1001, 84.8%). Interestingly, for the first time in the history of the Greek HIV-1 epidemic, most HIV-1 infections were reported among drug injectors (n=522, 44.2%), followed by men who have sex with men (n=304, 25.8%). The proportion of non-Greek nationals among HIV-1 infected cases was increased (n=254, 21.5%) compared to 2011. Among HIV-1 infected drug injectors, non-Greek nationals constituted almost one fourth of total (n=124, 23.8%), while among non-IDU infectees, there were 130 non-Greeks (19.8%). The Table S1 shows the temporal trends of IDUs by nationality.

Data on the place of residence were available for 78% of IDU cases reported in 2011. Among them, the majority (86%) were residents of the Metropolitan Area of Athens. In 2012, the number of HIV diagnoses among IDUs that were reported to the Greek health authorities doubled (n=522; 4.8 cases per 100,000 population) compared to 2011. Males (n=429, 82.2%) and Greek nationals (n=388, 74.3%) comprised the majority of HIV reports among IDUs in 2012. The place of residence was provided by 67.8% of HIV-1 IDUs reported in 2012 and 93.9% of them were living in the Athens Metropolitan Area at the time of HIV report.

*Phylogeography of HIV-1 sequences in IDUs*

To identify the geographic origin of the IDUs sequences from the 2 large clades of subtypes A and G, we performed a BLAST similarity search against all published HIV-1 sequences. Further phylogenetic analysis for subtype A including CRF35_AD isolates revealed that the sequences from the largest IDUs clade in Greece was part of the CRF35_AD branch spreading among IDUs in Iran and Afghanistan (Figure S1) while, the second largest IDUs cluster (19 sequences) was nested within the CRF14_BG, spreading in South-Western (SW) Europe (Portugal and Spain).

HIV-1 sequences according to nationality were: for the largest clade (CRF35_AD), 32 out of 36 subjects (88∙9%) were Greek nationals, 1 person originated from Iran (2∙8%), while for 3 the nationality was unknown (8∙3%). For the CRF14_BG, 17 out of 19 individuals were of Greek nationality (89∙5%), and the remaining 2 were nationals of Albania and Bulgaria. For subtype B, 10 out of 12 (83∙3%) persons were Greek nationals and 2 were from Afghanistan and Iraq. For the smallest clade of subtype A, 3 individuals were Greek nationals (60%), 1 (20%) was from Iran and for one individual the nationality was unknown.

*Phylodynamic analyses*

Molecular clock analyses revealed the time to most recent common ancestor (tMRCA). The tMRCA of a phylogenetic cluster is the time since the founding of the cluster or the time since the first transmission that lead to the formation of the observed clustered infections. It also provides the upper bound for the time of secondary infections within the members of the cluster .

Searching for potential founders shown in Figure S2, an isolate from an Iranian national appears as the potential founder for the CRF35_AD outbreak (Figure S2B). Similarly, the CRF14_BG epidemic was probably introduced from a Bulgarian national (Figure S2D), while the other groups were founded by Greek nationals and sequences from non-Greek nationals were nested within the IDUs clades of Greek nationals (Figure S2A).

**References**

1. Paraskevis D, Magiorkinis E, Magiorkinis G, Sypsa V, Paparizos V, et al. (2007) Increasing prevalence of HIV-1 subtype A in Greece: estimating epidemic history and origin. J Infect Dis 196: 1167-1176.

2. Paraskevis DM, E. Zavitsanou, A. Detsika, M. Magiorkinis, G. Papa, A. Beloukas, A. Nikolopoulos, G. Sypsa, V. Tsiara, C. Malisiovas, N. de Oliveira, T. Hatzakis, A. for the multicenter study of HIV heterogeneity. Molecular typing of the HIV-1 networks through a nationwide study in Greece: predominance of subtype A over B spreading among the natives; 2010; Amsterdam, The Netherlands.

3. Price MN, Dehal PS, Arkin AP (2010) FastTree 2--approximately maximum-likelihood trees for large alignments. PloS one 5: e9490.

4. Paraskevis D, Pybus O, Magiorkinis G, Hatzakis A, Wensing AM, et al. (2009) Tracing the HIV-1 subtype B mobility in Europe: a phylogeographic approach. Retrovirology 6: 49.

5. Drummond AJ, Rambaut A (2007) BEAST: Bayesian evolutionary analysis by sampling trees. BMC evolutionary biology 7: 214.

6. Drummond AJ, Ho SY, Phillips MJ, Rambaut A (2006) Relaxed phylogenetics and dating with confidence. PLoS biology 4: e88.

7. Sanders-Buell E, Saad MD, Abed AM, Bose M, Todd CS, et al. (2007) A nascent HIV type 1 epidemic among injecting drug users in Kabul, Afghanistan is dominated by complex AD recombinant strain, CRF35_AD. AIDS research and human retroviruses 23: 834-839.

8. Leitner T, Albert J (1999) The molecular clock of HIV-1 unveiled through analysis of a known transmission history. Proceedings of the National Academy of Sciences of the United States of America 96: 10752-10757.

9. Paraskevis D, Magiorkinis E, Magiorkinis G, Kiosses VG, Lemey P, et al. (2004) Phylogenetic reconstruction of a known HIV-1 CRF04_cpx transmission network using maximum likelihood and Bayesian methods. Journal of molecular evolution 59: 709-717.

10. Pharris A, Wiessing L, Sfetcu O, Hedrich D, Botescu A, et al. (2011) Human immunodeficiency virus in injecting drug users in Europe following a reported increase of cases in Greece and Romania, 2011. Euro surveillance : bulletin europeen sur les maladies transmissibles = European communicable disease bulletin 16.

**Table** S1. Number of reported injecting drug users (IDUs) by nationality status (Greek, non-Greek) during the years 2001-2012

|  | **Nationality** | | |  |
| --- | --- | --- | --- | --- |
| **Year** | **Greek** | **Non-Greek** | **Unknown** | **Total** |
| 2001 | 11  73∙3% | 4  26∙7% | 0 | 15  100∙0 |
| 2002 | 7  41∙2% | 10  58∙8% | 0 | 17  100∙0 |
| 2003 | 9  75∙0% | 2  16∙7% | 1  8∙3% | 12  100∙0 |
| 2004 | 6  54∙6% | 4  36∙4% | 1  9∙0% | 11  100∙0 |
| 2005 | 11  57∙9% | 7  36∙8% | 1  5∙3% | 19  100∙0 |
| 2006 | 10  58∙8% | 7  41∙2% | 0 | 17  100∙0 |
| 2007 | 7  63∙6% | 4  36∙4% | 0 | 11  100∙0 |
| 2008 | 7  63∙6% | 4  36∙4% | 0 | 11  100∙0 |
| 2009 | 9  64∙3% | 5  35∙7% | 0 | 14  100∙0 |
| 2010 | 8  53∙3% | 7  46∙7% | 0 | 15  100∙0 |
| 2011 | 211  81∙2 | 43  16∙5% | 6  2∙3% | 260  100∙0 |
| **2012** | 388  74∙3% | 124  23∙8% | 10  1∙9% | 522  100∙0 |
| **Total** | 684  74∙0% | 221  23∙9% | 19  2∙1% | 924  100∙0 |

**Table** S**2**. Distribution of clustered HIV-1 infections in injecting drug users (IDUs) per sampling year

| **Sampling year** | **Clustered HIV-1 infections** | **Non-clustered HIV-1 infections** | **Total** | **IDUs among HIV-1 reported cases** | **Percentage of reported IDUs with sequences[[1]](#footnote-2)** |
| --- | --- | --- | --- | --- | --- |
| 1998 | 0 | 5 | 5 | 15 | 33.3 |
| 1999 | 0 | 7 | 7 | 29 | 24.1 |
| 2000 | 0 | 10 | 10 | 21 | 47.6 |
| 2001 | 0 | 5 | 5 | 15 | 33.3 |
| 2002 | 2 | 3 | 5 | 17 | 29.4 |
| 2003 | 1 | 10 | 11 | 12 | 91.7 |
| 2004 | 0 | 12 | 12[[2]](#footnote-3) | 11 | 100.0 |
| 2005 | 1 | 2 | 3 | 19 | 15.8 |
| 2006 | 0 | 2 | 2 | 17 | 11.8 |
| 2007 | 0 | 6 | 6 | 11 | 54.5 |
| 2008 | 0 | 5 | 5 | 11 | 45.5 |
| 2009 | 0 | 5 | 5 | 14 | 35.7 |
| 2010 | 5 | 7 | 12 | 15 | 80.0 |
| 2011 | 106 | 6 | 112 | 260 | 43.1 |
| 2012 | 76 | 6 | 82 | 205^2^ | 40.0[[3]](#footnote-4) |

**Supporting Information Legends**

**Figure S1** Part of phylogenetic tree for subtype A sequences sampled from different areas [Africa, Albania, other European countries, former Soviet Union (FSU) areas, Afghanistan/Iran and Greece]. Different colors were used for the non-injecting drug users (IDUs) and IDUs sampled before and after 2010 from Greece.

**Figure S2** Partial dated phylogenetic trees showing the ethnic origin of the individuals from whom HIV-1 sequences were sampled. For two sub-outbreaks (CRF35_AD and CRF14_BG) the potential founders were non-Greek nationals, while for subtype A and subtype B (A and C) the potential founders were nationals.

**Figure S3a** Trends in the estimated number of syringes distributed / exchanged through specialized programmes per estimated injecting drug user (IDU) in the city of Athens (NSP coverage, 2004-2011).

**Figure S3b** Opioid substitution treatment (OST) coverage per estimated problem drug user (addicted to opioids) during the years 2002-2011. Data were not collected in 2007.

1. Percentage of IDUs sequenced with regard to the number of HIV-1 reported among IDUs every year. HIV-1 sequences are reported according to sampling year [↑](#footnote-ref-2)
2. **Sequences from IDUs are reported according to sampling year and not year of diagnosis. This is the reason for the discrepancy observed for year 2004.** [↑](#footnote-ref-3)
3. For 2012 samples were collected between January-May 2012. Similarly reported cases correspond to the IDUs diagnosed with HIV-1 infection until 31/5/2012. [↑](#footnote-ref-4)
